# The benefit of evolving a larger brain: big-brained guppies perform better in a cognitive task^☆^

**DOI:** 10.1016/j.anbehav.2013.07.011

**Published:** 2013-10

**Authors:** Alexander Kotrschal, Björn Rogell, Andreas Bundsen, Beatrice Svensson, Susanne Zajitschek, Ioana Brännström, Simone Immler, Alexei A. Maklakov, Niclas Kolm

**Affiliations:** a Animal Ecology, Department of Ecology and Genetics, Uppsala University, Uppsala, Sweden; b Evolutionary Biology, Department of Ecology and Genetics, Uppsala University, Uppsala, Sweden

**Keywords:** artificial selection, brain size, cognition, guppy, learning, *Poecilia reticulata*

## Abstract

• We previously selected for large and small brain size in guppies.
• Large-brained females outperformed small-brained females in a learning task.
• Healy and Rowe challenged our interpretations of larger brains = better learning.
• Here we argue why we think they are mistaken.

Vertebrate brain size is thought to evolve through a balance between positive selection on cognitive abilities and evolutionary costs (Striedter 2005). In a recent study we tested this hypothesis experimentally by investigating the costs and benefits of evolving a larger brain (Kotrschal et al. 2013). We selectively bred guppies, *Poecilia reticulata*, for large and small brain size relative to body size and found that brain size evolved rapidly in response to divergent selection. After two generations of selection, large-brained females outperformed small-brained females in a learning task that we used to test their ability of numerical associative learning. The costs of evolving a larger brain size were apparent since large-brained fish developed smaller guts and produced fewer offspring. Thus, our study was the first to demonstrate experimentally that evolving a larger brain comes at the cost of a decreased gut mass and decreased offspring production, while conferring a cognitive advantage. Apart from identifying costs of evolving a larger brain we thus provided direct experimental evidence for what have been previously suggested by a large number of comparative studies (Gittleman 1994; Lefebvre et al. 1997; Pravosudov & Clayton 2002; van Schaik & Pradhan 2003; Garamszegi & Eens 2004; Tebbich & Bshary 2004; Sol et al. 2007; Gonzalez-Voyer et al. 2009; Herculano-Houzel 2009; Kotrschal et al. 2012a, b), but doubted by others (Healy & Rowe 2007): that relative brain size is indicative of an animal's cognitive ability.

Healy & Rowe (2013) challenge our interpretation of the learning assay in the guppy experiment and suggest alternative explanations for our results. In the following we explain why we think they are mistaken.

First, Healy & Rowe suggest that a potential discrepancy in the stimulus–reward contingency between fish of large and small relative brain size might provide an alternative explanation for why large-brained females outperformed small-brained females. When training our fish we placed cards with either four or two symbols on either side of the individual holding tanks and provided food only on the side of the tank with the number of symbols to which the fish was trained. The fish were left to forage, find the food and over time learn to connect the correct number of symbols with the food reward. When we tested whether they had learnt that a certain number of symbols was connected to a food reward, we provided the cards without food and noted to which side individuals would swim first. As Healy & Rowe correctly state, for visual discrimination learning ‘the fish must have sampled both the rewarded and unrewarded stimuli during training’. Along those lines they suggest that the small-brained fish might not have fed during the trials and therefore not have had the chance to learn to associate the correct stimuli with the food reward. This argument is not valid because we know that all fish fed every day throughout the trials: throughout the experiment, the food given during training and testing was the only food available to the fish. We provided a standard (more than usually eaten by one fish within 90 min) quantity of food and removed leftover food after every trial. We therefore had perfect control over whether a fish had fed or not. In fact, in another experiment we found no difference between the feeding propensity of large- and small-brained females (*P* = 0.176; Fig. 1; also note that the nonsignificant effect is in the opposite direction to that predicted by Healy & Rowe). Therefore, differences in feeding propensities between large- and small-brained females are unlikely to have biased the outcome of the experiment as suggested by Healy & Rowe.

Second, Healy & Rowe question whether we adequately excluded a potential inherent preference for four versus two symbols in the large-brained females because we tested for this preference in another group of fish than the one used for the learning task. Healy & Rowe suggest that, while a preference in the control fish may not exist, we ‘cannot be sure that those fish used in the learning task were free from biases’. This remark stems from a misunderstanding of our experimental design. The key point here is that we had three replicate populations of each of our selection regimes (small or large brains). By randomly sampling fish from each of these replicate populations, and including replicate population as a random effect in every analysis, we ensured that the observed differences between the selection regimes reflected hereditary properties associated with the selection regimes. In our case, the trained large-brained guppies outperformed the trained small-brained guppies (*P* = 0.006). We tested whether this could be explained by a preference for a specific number of symbols associated with brain size by testing another random sample from the very same replicate populations as in the learning trials, but without the training. Despite the same number of individuals being tested, we found no such pattern (*P* = 0.192), and concluded that brain size-mediated preference for a specific number of symbols is an unlikely explanation for the observed patterns. Healy & Rowe's assertion of unlearned bias in our trials is based on the expectation that one random subsample of a population would show a bias towards four symbols, while another random subsample of the same population would not show such a bias across three independent replicate lines. This is essentially the null hypothesis that we tested and refuted in our paper.

Third, Healy & Rowe raise the concern that motivational differences from differential energy requirements between fish with large and small brain size may have affected our results. They argue that large-brained females need to feed more to maintain their energetically costly large brains, and hence have more opportunity to learn the rewarded stimulus. However, as mentioned above (Fig. 1), large-brained females do not differ in their feeding propensity, and it is possible that the guppies solved their energy demands by reallocating resources to brain development, as we showed in our paper. Indeed, theoretical and comparative studies suggest such a change in energy allocation during brain size evolution (Aiello & Wheeler 1995; Kaufman et al. 2003; Naya et al. 2007). Assays of metabolic rate, conversion efficiency and feeding requirement in the brain size-selected guppies are currently underway to investigate this issue further.

In their fourth point, Healy & Rowe question the stimulus characteristics used in the experiment. We trained our fish on four small (0.25 cm^2^ each) versus two large (0.5 cm^2^ each) symbols, but then tested them on four versus two symbols of equal sizes (0.375 cm^2^). Such a design is an integral part of most standard tests of numerical learning ability in fish to ensure that fish would choose the correct stimulus based on the numerical concept of two versus four and not according to symbol size (Agrillo et al. 2007). Healy & Rowe's concerns could apply to a null result, but cannot explain the actual differences found in our experiment.

Finally, Healy & Rowe suggest that differences in performance might have been caused by differences in salience. We agree that this is an interesting question but we think the argumentation provided by Healy & Rowe is contradictory and does not follow the current understanding of the evolution of separate brain regions. On the one hand, Healy & Rowe argue that visual cues may be more salient to large-brained animals because their optic tectum (the part of the brain that processes visual information) is larger than the optic tectum of small-brained fish. On the other hand, they suggest that small-brained females may rely more on olfaction to find food, thereby ignoring visual symbols and not learning the discrimination task. This argument seems inconsistent to us. Why should an overall increase in relative brain size lead to a shift towards preferentially relying on optic information, but a decrease in relative brain size lead to a shift towards relying on olfaction? A shift of sensory capabilities is usually accompanied by a change in relative size of the region responsible for processing the information of the respective organ (K. Kotrschal et al. 1998; Striedter 2005; A. Kotrschal et al. 2012b). But, as Healy & Rowe correctly state, the regions of the brain are not different in size between large- and small-brained guppies (Kotrschal et al. 2012a). If a shift had occurred we would have expected a corresponding change in relative region size.

We agree with Healy & Rowe that the demonstration of a cognitive advantage to having a larger brain is of ‘major significance for understanding the relationship between brain size and cognitive abilities’ because it gives ‘much needed empirical support to the assumption that the one is a good proxy of the other’. We therefore continued to investigate the cognitive abilities of large- and small-brained fish in diverse assays. For example, to increase the motivation of male guppies to participate in a learning trial in a recent experiment we rewarded them with a mating opportunity instead of food and found that large-brained males were faster to learn their way through a complex maze to find the females (Kotrschal et al., unpublished data). This result highlights important motivational differences between the sexes: while food intake was of high importance for the females, the acquisition of mates was the driving force for males. The cognitive advantage of a bigger brain was thus unequivocally revealed, in both sexes, by offering the rewarding stimulus that reflected motivation.

We welcome the interest in our paper and remain confident in our original conclusion that relatively bigger brains really are better in cognitive terms. Although we certainly appreciate the need for future studies on the finer details of brain morphology to understand the link between brain morphology and cognition, we maintain that brain size can be considered a good proxy for cognitive ability and further note that this is not really a controversial issue in light of the extensive literature supporting a positive association between brain size and cognitive ability (e.g. Darwin 1871; Gittleman 1994; Lefebvre et al. 1997; Pravosudov & Clayton 2002; van Schaik & Pradhan 2003; Garamszegi & Eens 2004; Tebbich & Bshary 2004; Sol et al. 2007; Gonzalez-Voyer et al. 2009; Herculano-Houzel 2009; Kotrschal et al. 2012a, b). The great pioneer in our field, Charles Darwin, had surprisingly insightful thoughts on many of the modern issues in evolutionary biology and this is the case also this time. We therefore wish to end with a fitting quote:

No one, I presume, doubts that the large proportion which the size of man's brain bears to his body, compared to the same proportion in the gorilla or orang, is closely connected with his higher mental powers (**Darwin 1871****, page 54**)

## Figures and Tables

**Figure 1:**
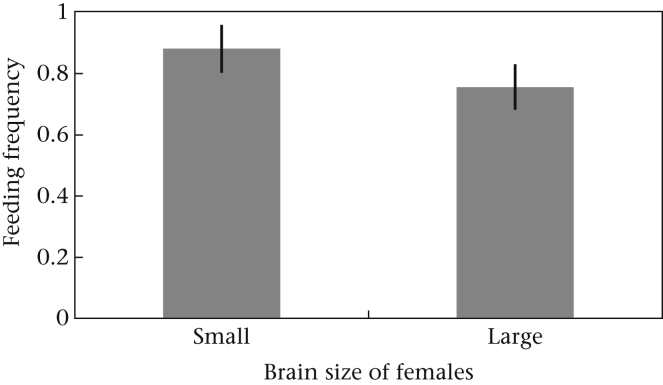
Feeding propensity of female guppies selected for large and small brain size when offered a novel food source (a pellet instead of flake food: 0 = never, 1 = always). Food was given once per day for 7 consecutive days. Feeding propensity is the number of times the fish ate the pellet (out of seven times). To analyse feeding propensity we used a binary general linear mixed model with feeding (yes/no) as the dependent variable, brain size treatment as a fixed factor, and replicate line, day of feeding and individual as random factors (GLMM: *F* = 1.945, *N* = 24, *P* = 0.176; the figure shows the mean estimated marginal means ± SE of this GLM).
